# Neural Correlates of Indicators of Sound Change in Cantonese: Evidence from Cortical and Subcortical Processes

**DOI:** 10.3389/fnhum.2016.00652

**Published:** 2016-12-23

**Authors:** Akshay R. Maggu, Fang Liu, Mark Antoniou, Patrick C. M. Wong

**Affiliations:** ^1^Department of Linguistics and Modern Languages, The Chinese University of Hong KongHong Kong, China; ^2^School of Psychology and Clinical Language Sciences, University of ReadingReading, UK; ^3^The MARCS Institute for Brain, Behaviour and Development, Western Sydney UniversityPenrith, NSW, Australia; ^4^Brain and Mind Institute, The Chinese University of Hong KongHong Kong, China; ^5^The Chinese University of Hong Kong—Utrecht University Joint Center for Language, Mind and BrainHong Kong, China

**Keywords:** language change, phonetics, individual variability, brainstem encoding, neurophysiology

## Abstract

Across time, languages undergo changes in phonetic, syntactic, and semantic dimensions. Social, cognitive, and cultural factors contribute to sound change, a phenomenon in which the phonetics of a language undergo changes over time. Individuals who misperceive and produce speech in a slightly divergent manner (called *innovators*) contribute to variability in the society, eventually leading to sound change. However, the cause of variability in these individuals is still unknown. In this study, we examined whether such misperceptions are represented in neural processes of the auditory system. We investigated behavioral, subcortical (via FFR), and cortical (via P300) manifestations of sound change processing in Cantonese, a Chinese language in which several lexical tones are merging. Across the merging categories, we observed a similar gradation of speech perception abilities in both behavior and the brain (subcortical and cortical processes). Further, we also found that behavioral evidence of tone merging correlated with subjects' encoding at the subcortical and cortical levels. These findings indicate that tone-merger categories, that are indicators of sound change in Cantonese, are represented neurophysiologically with high fidelity. Using our results, we speculate that *innovators* encode speech in a slightly deviant neurophysiological manner, and thus produce speech divergently that eventually spreads across the community and contributes to sound change.

## Introduction

Language is a dynamic and adaptive system that undergoes diachronic and synchronic changes (Beckner et al., 2009). Some of the well-documented changes across languages include the great vowel chain shifts between the 14th and 16th century (Wolfe, 1972), phonological mergers in Sinitic languages (Shen, 1997) and lexical borrowing between languages (Bloomfield, 1933; Cheng, 1987). In addition to documenting change, research studies have also investigated the sources of change, predominantly by examining the impact of social factors (Labov, 1980, 1990, 2006; Haeri, 1996; Eckert, 2000) on language. In recent years, a new set of studies have begun to focus on speaker-internal factors that could drive change, including individual differences in perceptual and cognitive processes (Witkin et al., 1975; Messick, 1976; Ausburn and Ausburn, 1978; Labov, 2001; Yu, 2010, 2013; Browman and Goldstein, 2014). The aim of the current study is to further investigate speaker-internal factors by examining how individual differences in neurophysiological processing may be associated with language change. The sound system of Cantonese is undergoing a course of change in which some speech categories are being merged. Most interestingly, the extent of merging differs across individuals and across speech categories. This gives rise to the opportunity for the current study to connect the extent of merging to differences in neurophysiological responses across the auditory neural pathway.

For decades, a large body of influential work on language change by Labov and others has found social factors to be predominant driving forces of change. Among the factors investigated are the effects of local communities (Eckert, 2000), socio-economic classes (Trudgill, 1974; Weinberg, 1974; Labov, 1980, 2006; Haeri, 1996; Velde et al., 2001), gender (Gauchat, 1905; Hermann, 1929; Labov, 1990, 2001; Yu, 2010), regional dialect (Labov, 2010), and frequency of usage (Hooper, 1976; Pierrehumbert, 2001; Abramowicz, 2007; Zhao and Jurafsky, 2009). Apart from social factors, cognitive differences among the individuals have also been considered as important contributors toward sound change (Ohala, 1990; Labov, 2010). It has been postulated that in a society, there are *innovators* who are individuals with a slightly deviant style of speech perception and production, and *early adopters* who adopt their deviant style (Milroy and Milroy, 1985). As *innovators* and *early adopters* interact, there is a spread of divergent speech characteristics in the society that during the course of time leads to modification of norm ultimately leading to sound change. Research studies also ask what speaker-internal factors may lead certain individuals in society to become *innovators* and others *early adopters*. Perceptual and cognitive factors are likely to contribute to such biases (Yu, 2010, 2013). In particular, individual differences in speech perception can be a particularly important driving force (Yu, 2010; Ou et al., 2015). As certain individuals misperceive speech, the misperception could lead to adjustments in production norms leading to development of new variants. When these variants are associated with social significance and spread to the rest of the speech community, sound change occurs. Ohala (1993) reports that the three main mechanisms responsible for sound change are hypocorrection in which a listener under-corrects coarticulatory effects; hypercorrection in which a listener overcorrects coarticulatory effects; and confusion between the similar acoustical sounds that occurs due to failure of a listener to retrieve a feature in one sound but not in the other. One possibility is that the listeners who hyper/hypocorrect are the *innovators* who perceive and produce in a divergent manner and thus, modify the norms of the community.

Are these confusions or misperceptions represented at the subcortical and cortical levels? For speech to be perceived, it must undergo stages of processing in both the peripheral (outer, middle and inner ear) and central (auditory nerve to brainstem to the cortex) components of the auditory pathway. The auditory brainstem and cortex are interconnected via numerous afferent and efferent neuronal pathways. The brainstem is a complex hierarchical structure consisting of cochlear nucleus, superior olivary complex, lateral lemniscus, and inferior colliculus interconnected via afferent, efferent, ipsilateral, and/or contralateral nerve fibers (Chandrasekaran and Kraus, 2010). The brainstem acts as a hub for auditory processing, and its activity can be measured using the frequency following response (FFR). FFR has been used as a metric of auditory system plasticity due to language experience (Krishnan et al., 2005, 2009; Swaminathan et al., 2008) and musical training (Musacchia et al., 2007, 2008; Wong et al., 2007; Bidelman et al., 2009). Furthermore, the brainstem with its sharp phase-locking characteristics (Galbraith et al., 1995) and top-down feedback from the cortex via efferent pathways, acts as an active contributor rather than a passive relay station in speech sound processing (Chandrasekaran et al., 2014). It has been found that clinical populations with disorders of speech, language and reading have impaired subcortical representation of sounds (Wible et al., 2005; Hornickel et al., 2009; Russo et al., 2009, 2010; Anderson et al., 2013). Given the importance of brainstem in speech sound encoding, we used FFR as a tool to study the distinction of tone categories in the brainstem. A deficiency in brainstem encoding may lead to defective representation of sounds in the cortex and vice versa (Chandrasekaran et al., 2014).

Apart from subcortical measures, cortical measures of auditory processing have also been found to be sensitive in determining the effects of long-term language experience (Buchwald et al., 1994; Chandrasekaran et al., 2007, 2009a; Zheng et al., 2012). Chandrasekaran et al. (2007) used mismatched negativity (MMN) and found that Chinese and English speakers differed on their pre-attentive ability to discriminate Mandarin lexical tone pairs. Further, Chandrasekaran et al. (2009a) also found that Chinese speakers processed lexical tones more faithfully than musicians. They attributed these findings to their long-term language experience. Though the effects of linguistic experience can be gauged with MMN or even earlier components, they may be driven by experience-dependent acoustic features (Maiste et al., 1995; Chandrasekaran et al., 2007, 2009b) rather than perception. In contrast, P300 is known to indicate discrimination of speech sounds based on phonological information (Maiste et al., 1995; Frenck-Mestre et al., 2005). Because of different attentional requirements, MMN and P300 index different stages of processing in the brain. MMN is a result of automatic change detection regardless of whether the change occurs in the acoustic or phonemic domain (Näätänen et al., 2007) and is a relatively early-stage phenomenon occurring in the latency range of 100–300 ms. P300, in contrast, is an event related potential (ERP) usually elicited in the range of 250–500 ms in an oddball paradigm where attention needs to be allocated to the deviant stimuli. In a P300 task, memory schemas are built with the incoming standard stimuli and every time a rare or deviant stimulus appears, these schemas are updated. P300 amplitude and latency is usually modulated by arousal levels and the amount of effort required in detection of the deviant stimuli (Polich, 2007). The more effortful the task, the longer the P300 latency and the higher the amplitude (Luck, 2005). P300 has been used to study the effects of linguistic experience (Buchwald et al., 1994; Zheng et al., 2012). Buchwald et al. (1994) studied whether Japanese listeners could differentiate/r/ and /l/ phonemes that are contrasted in English but not in Japanese. They found that adult native speakers of Japanese had reduced P300 for detecting [r] and [l] sounds. Zheng et al. (2012) found that Cantonese speakers had enhanced P300 amplitudes relative to their Mandarin counterparts on a lexical tone oddball discrimination task, mainly due to long-term experience with the more complex tone space in Cantonese. As categorical perception of lexical tones can be well represented neurophysiologically using P300 (Zheng et al., 2012), we used this ERP technique in the current study to probe active language processing at the cortical level. In sum, we employed a combination of FFR and P300 to probe passive (subcortical) and active (cortical) levels of auditory processing to investigate neural representations of the phenomenon of sound change.

It is known that encoding at the auditory brainstem is shaped by language experience and tone language speakers encode lexical tones more robustly than non-tone language speakers (Krishnan et al., 2005, 2009; Swaminathan et al., 2008). Krishnan et al. (2005, 2009, 2010) argue that local mechanisms in the brainstem circuitry show plasticity toward long-term experience. On the other hand, there is also evidence that brainstem encoding is driven by top-down cortical feedback (Suga, 2008; Bajo et al., 2010). Recently, it has been found that the effects visible at the brainstem originate at the level of cortex and are driven via corticofugal pathways from cortex to the brainstem (Suga, 2008; Parbery-Clark et al., 2009; Krizman et al., 2012; Song et al., 2012; Hairston et al., 2013; Skoe and Kraus, 2013). As literature supports the effect of tone language experience both at the brainstem and cortex, it is important to probe these levels of the auditory pathway using FFR and P300 in order to understand whether sound change is represented at the brainstem and/or cortical level.

In order to investigate the neural representations of sound change in the auditory pathway, we examined the neural responses to speech categories in Cantonese that are undergoing various degrees of merging. Cantonese is a Chinese language that has six lexical tones (high-level, mid-level, low-level, high-rising, low-rising, and falling), which are pitch patterns used to distinguish word meanings (e.g., the syllable /ji/ means “chair” when spoken with high-rising tone 2 and “ear” when spoken with low-rising tone 5). What makes Cantonese particularly useful for studying the neural basis of sound change is that some of the tone categories are in the process of undergoing merging to various degrees. Merging leads to the disappearance and emergence of speech sound categories in a language. The merging phenomenon may be observed in the English of some speakers in the southern United States where they have lost distinction between “pen” and “pin” (the sound “e” has been lost) (Labov et al., 2005). In Cantonese, several degrees of merging can be observed across the tone categories. Degrees of merging are determined based on the prevalence of confusions in native Cantonese speakers' perception and/or production (Law et al., 2013; Mok et al., 2013). The two rising lexical tones of Cantonese, Tones 2 and 5 (denoted by the symbols T2 and T5, respectively) are confused most often and thus constitute a *full-merger* pair, followed by Tones 4 (falling pitch) and 6 (low-level pitch) forming a *near-merger* pair (T4/T6), and Tones 3 (mid-level pitch) and 6 (low-level pitch) form a *quasi-merger* pair (T3/T6) with the least confusion compared to the other merging pairs (Fung and Wong, 2011). Conversely, Tones 1 (high-level pitch) and 2 (high-rising pitch) form a non-merger pair (T1/T2) causing no confusion in perception. Further, lexical tones are especially useful for investigating subcortical auditory processing because the brainstem response is phase-locked to the fundamental frequency (F0), the acoustic correlate of lexical tone.

In the current study, we explored the neural representations of sound change by investigating tone merging at behavioral, brainstem and cortical levels. Given that perceptual and cognitive factors are thought to contribute to sound change (Yu, 2010, 2013), we hypothesized individual variability in the behavioral, brainstem and/or cortical encoding of speech sounds. Specifically, we predicted a high correlation between the behavioral and neurophysiological manifestations of merging. Additionally, we predicted a similar gradation of degree of tone merging in the behavior and brainstem/cortical representations.

To evaluate tone merging at the level of the brainstem, we recorded FFR from adult native Cantonese speakers (*n* = 30) using the six lexical tones of Cantonese. The magnitude of merging in the brainstem was determined using response-to-response correlation of FFR pitch contours of the tones in the relevant tone pairs (T1/T2, T2/T5, T3/T6, T4/T6). Higher correlations between FFR pitch contours would indicate a greater degree of merging in the brainstem. For evaluating cortical correlates of tone merging, we administered P300 in an oddball paradigm using blocks of relevant tone pairs (T1/T2, T2/T5, T3/T6, T4/T6) in adult native Cantonese speakers (*n* = 25) and analyzed the amplitude and latency. P300 amplitude/latency depends on the amount of effort a listener devotes to a task (Luck, 2005). In the current study, merging pairs are expected to be more effortful than non-merging pairs resulting in higher P300 amplitude and latency. Both brainstem and cortical measures of tone merging were analyzed alongside behavioral discrimination scores obtained from all the participants in order to understand the individual variability in neurophysiology contributing to sound change.

## Materials and methods

Two experiments were carried out in the current study. The first explored the subcortical representation of tone merging using FFR, and the second examined cortical representation of tone merging using P300. Additionally, behavioral data were collected in both experiments using AX discrimination tasks.

### Stimuli for neurophysiological data acquisition

**Figure 1:**
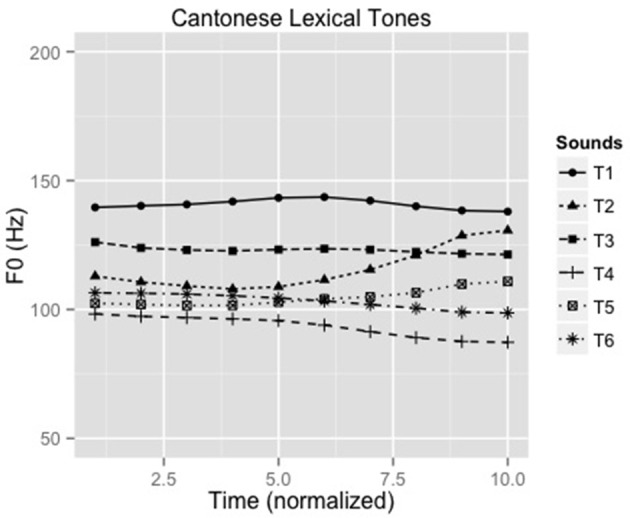
**F0 contours of all six Cantonese tones (F0 ranges: T1:135-146 Hz, T2: 105-134 Hz, T3: 120-124 Hz, T4: 85-99 Hz, T5: 98-113 Hz, T6: 98-106 Hz, respectively)**.

A 25-year old male native speaker of Cantonese, phonetically-trained for production of Cantonese lexical tones, produced the syllable /ji/ with six Cantonese lexical tones making six unique words (Liu et al., 2015): /ji1/ “doctor,” /ji2/ “chair,” /ji3/ “meaning,” /ji4/ “son,” /ji5/ “ear” and /ji6/ “justice.” Figure 1 shows the contour of the six lexical tones of Cantonese (F0 ranges: 135–146, 105–134, 120–124, 85–99, 98–113, 98–106). Recordings were conducted in an acoustic booth using a Shure SM10A microphone and Praat (Boersma and Weenink, 2010) with a sampling rate of 44.1 kHz. Five versions of the stimuli were created with durations normalized to 150, 175, 200, 225, and 250 msec. A speech identification task was administered on 12 native Cantonese listeners to select the stimuli with the most appropriate stimulus duration for the experiment. The 175 ms stimuli were consistently and correctly identified and thus were used as stimuli for the experiment.

### Participants

Thirty native speakers of Cantonese (*M* age = 21.7 years; range = 18–29 years; 10 males), studying at the Chinese University of Hong Kong, participated in the FFR experiment. Of these, seventeen had some formal musical training (*M* years of training = 3.1; range = 1–13 years). Twenty-five participants (*M* age = 23.0; range = 21–27 years; 6 males), studying at the Chinese University of Hong Kong participated in the P300 experiment. Of these, sixteen had some formal musical training (*M* years of training = 3.2; range = 1–12 years). All participants had peripheral hearing sensitivity within 25 dB HL for 0.5–4 kHz. Written informed consents were obtained from all participants prior to the experiments. The Joint Chinese University of Hong Kong—New Territories East Cluster Clinical Research Ethics Committee approved the study.

### Procedure

#### Experiment 1

##### FFR acquisition

Brainstem responses collected from 3000 sweeps of each stimulus presented in alternating polarity were added to minimize stimulus artifacts and cochlear microphonics (Gorga et al., 1985; Skoe and Kraus, 2010). Stimuli were routed to the right ear through ear inserts (Compumedics 10 Ω) at 81 dB SPL with an interstimulus interval (offset to onset) jittered between 74 and 104 msec (Wong et al., 2007; Liu et al., 2015) using the Audio CPT module of STIM2 (Compumedics, USA). The order of stimulus presentation was randomized across participants. Responses were differentially collected from Ag/AgCl electrodes at Cz referenced to linked M1 and M2 (linked mastoids), with lower forehead as ground (Liu et al., 2015), using Synamps RT amplifier connected to Curry 7.05 workstation (Compumedics, El Paso, TX). Inter-electrode impedances were maintained at less than or equal to 1 kΩ. The responses were collected at a sampling rate of 20 kHz. Offline analysis consisting of artifact rejection, filtering, epoching, averaging, converting to wave files was done using Curry 7.05 (Compumedics, El Paso, TX). Sweeps with voltage beyond ± 35 μV were considered artifacts and rejected. The waveforms were band-pass filtered from 80 to 5000 Hz (with 6 dB roll off) with a 275 msec time window consisting of 50 msec pre-and post-stimulus baselines. The EEG recordings with more than 10% of the sweeps rejected (i.e., >300 rejections), were not included in further analyses.

##### FFR analysis procedures

**Figure 2:**
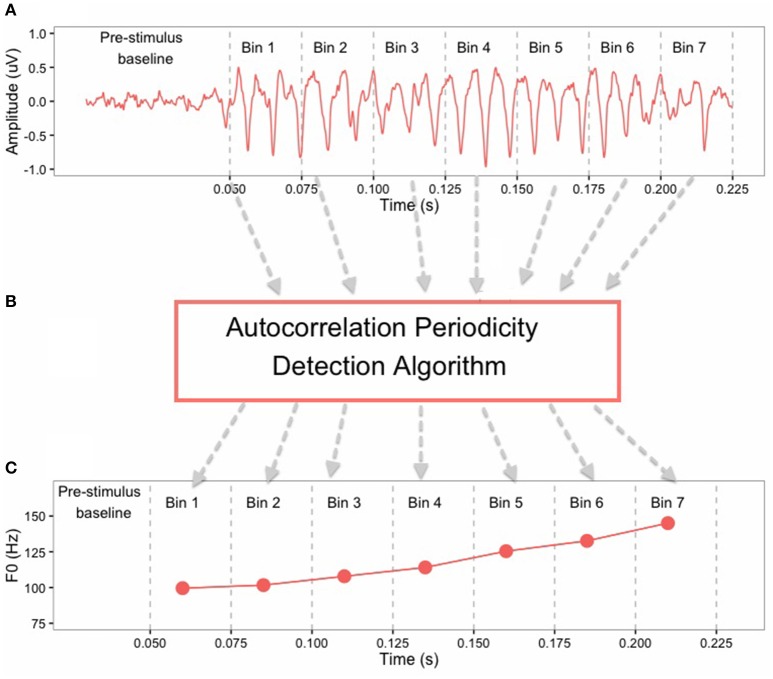
**Illustration of the method of extraction of F0 contour from FFR of a Cantonese lexical Tone 2 (T2) stimulus: (A)** the FFRs in the time-domain are divided into 7 bins of 25 ms each; **(B)** Autocorrelation periodicity detection algorithm works on each bin separately; **(C)** F0 points are obtained at each bin that are connected to form the F0 contour of the FFR.

In order to test phase-locking ability of the auditory brainstem, FFRs were band-pass filtered offline from 80 to 2500 Hz. Pitch contours of the FFRs were obtained using a periodicity detection autocorrelation algorithm in MATLAB 8.3 (The MathWorks, Inc, Natick, MA, USA). The FFR waveforms were converted from the temporal domain to the spectral domain and amplitude peaks around the F0 were extracted from seven non-overlapping 25-ms bins (0–25, 26–50, 51–75, 76–100, 101–125, 126–150, 151–175) (Krishnan et al., 2005). The F0 pitch contour of the FFR was constructed by connecting these peaks extracted from each time bin (Figure 2). Supplementary Figure 1 shows averaged FFR pitch contours from all subjects. The FFR pitch contours were used to calculate *response-to-response* correlation for each subject, which is a Pearson correlation between pitch contours (F0) of two brainstem responses. Here, response-to-response correlation serves as an indication of perceptual merging of the tone categories in brainstem and was calculated for the following tone pairs: T2/T5 (full-merger), T4/T6 (near-merger), T3/T6 (quasi-merger), and T1/T2 (non-merger). Strong positive response-to-response correlation values reflect a greater degree of merging for lexical tone pairs in the brainstem, whereas weak and/or negative correlations represent the absence of tone merging. Supplementary Tables 1, 2 provide correlation matrices for *stimulus-to-stimulus* and *response-to-response* correlations, respectively, for all possible tone-pair combinations.

##### Behavioral testing and analyses

**Figure 3:**
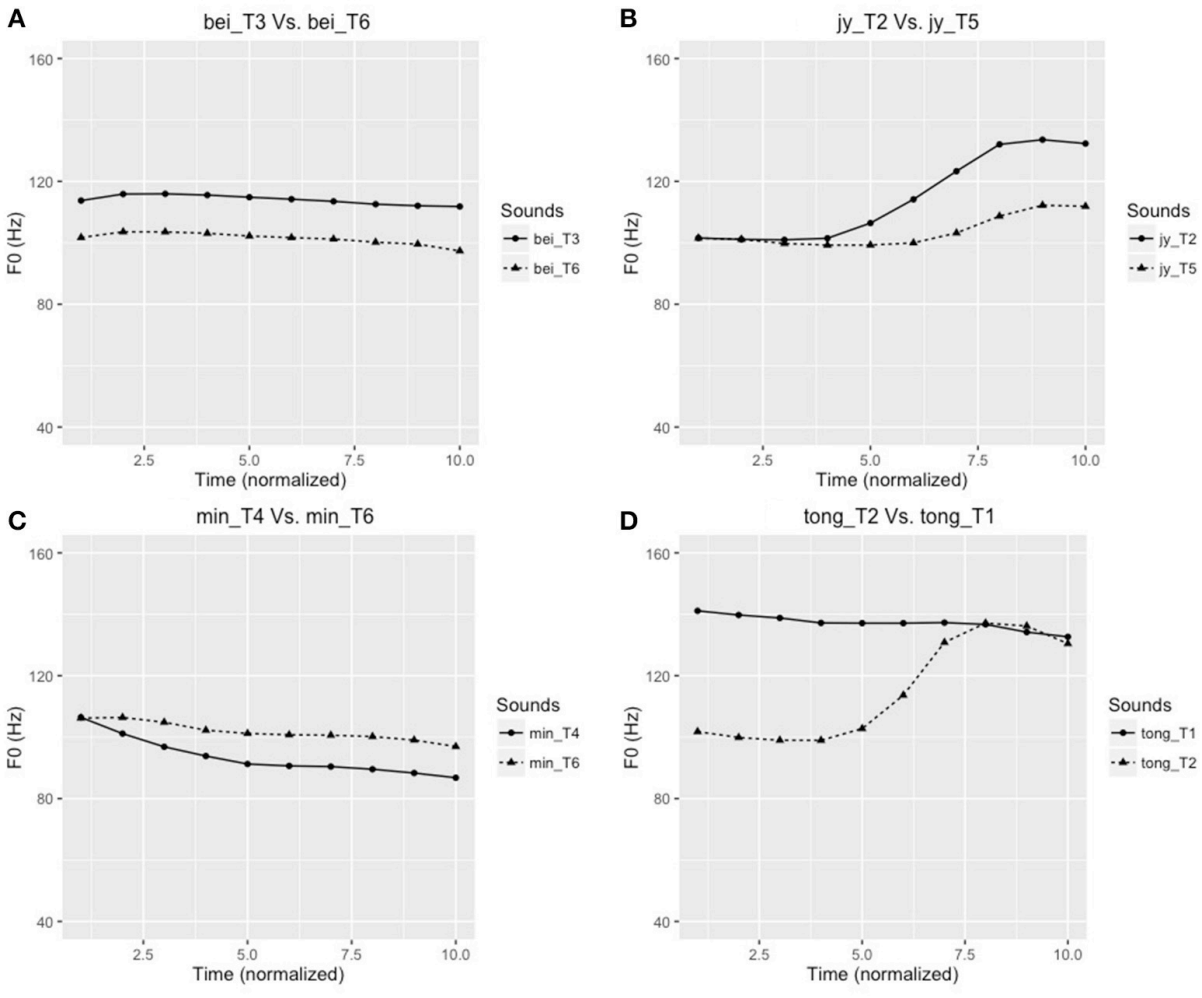
**Time-normalized F0 contours (in Hz) of the four tone pairs used in the AX discrimination task: (A)** bei_T6 [鼻,“nose”] – bei_T3 [臂,“arm”]; **(B)** jy_T5 [雨,“rain”] –jy_T2 [鱼,“fish”]; **(C)** min_T4 [綿,“cotton”] – min_T6 [麵,“noodle”]; **(D)** tong_T2 [糖,“sweet (candy)”] – tong_T1 [湯,“soup”]. Note: T1, T2, T3, T4, T5, and T6 correspond to the Cantonese high-level (Tone 1), high rising (Tone 2), mid-level (Tone 3), low-falling (Tone 4), low-rising (Tone 5), and low-level (Tone 6) tones, respectively.

An AX discrimination task was used to test the four tone pairs (T2/T5, T4/T6, T3/T6, T1/T2). To enhance ecological validity, the speech discrimination task used words different from those used in the FFR experiment. These words were “tong” (T2/T5), “jy” (T4/T6), “bei” (T3/T6), and “min” (T1/T2), selected from a subset of stimuli from Wong et al. (2009). The stimuli were normalized for duration and intensity, and differed only in their pitch contours (Figure 3). Four practice trials (with 5 repetitions), different from the experimental items were provided to the participants to familiarize them with the task. Participants were presented with a total of 80 stimulus pairs (4 tone pairs × 5 repetitions × 2 “identical” sequences × 2 “different” sequences) with an interstimulus interval of 500 msec. The participants were required to press the appropriate button to indicate “same” or “different” for the pitch patterns of the tone pairs. For each lexical tone pair, discrimination scores were recorded, and d′ scores were further calculated by subtracting z-scores of hits from false alarms (d′ = Z_FA_–Z_Hit_). This measures the subjects' discrimination sensitivity while accounting for response bias.

#### Experiment 2

##### P300 acquisition and analysis

P300 data were collected for the four tone pairs: Tone 2/Tone 5 (T2/T5; full-merger), Tone 3/Tone 6 (T3/T6; quasi-merger), Tone 4/Tone 6 (T4/T6; near-merger), Tone 1/Tone 2 (T1/T2; non-merger). For P300 acquisition, we collected averaged EEG responses from 1000 sweeps of each pair of stimuli in an oddball paradigm (Standard: Rare: 80: 20). The order of stimuli (tone pairs) and their presentation (standard and rare stimuli) were counterbalanced across subjects. The stimuli were presented at an intensity of 80 dB SPL to the right ear via insert ER-3A insert earphones (Etymotic research) at a repetition rate of every 0.85 s. Participants were instructed to press the response button as soon as they heard a deviant stimulus. As we were interested in only looking at the temporal aspects, EEG data were collected with a 4-electrode Cz-(M1+M2)-ground montage using an Intelligent Hearing Systems unit (Miami, USA). The responses were filtered from 1 to 30 Hz, rejected for artifacts beyond ± 75 μV, baseline corrected and averaged over a time window of −100 msec (pre-stim baseline) to 500 ms. The P300 peaks were identified as the maximum peak amplitude on the waveforms elicited by the deviant stimuli in the latency region of 250 to 400 ms. The peaks were identified independently by two experienced EEG researchers and in case of a disagreement, opinion of a third rater was sought.

##### Behavioral data collection

Given the technical constraints of the EEG instrument used, the behavioral responses could not be recorded simultaneously and thus, were collected separately. In order to test the discrimination ability of the participants for the 4 tone pairs (T2/T5, T4/T6, T3/T6, T1/T2) used in the P300 task, an AX discrimination task was constructed using the same set of stimuli used in P300 recording (/ji1/ to /ji6/). Participants were presented with a total of 48 stimuli pairs (4 tone pairs × 3 repetitions × 2 “identical” sequence × 2 “different” sequence) with an interstimulus interval of 500 ms. The participants were required to press the appropriate button to indicate “same” or “different” for the pitches of the tone pairs. For each lexical tone pair, accuracy, d′ (Z_FA_–Z_Hit_) and reaction time were calculated.

## Results

### Experiment 1: brainstem and behavior

#### Behavioral results

**Figure 4:**
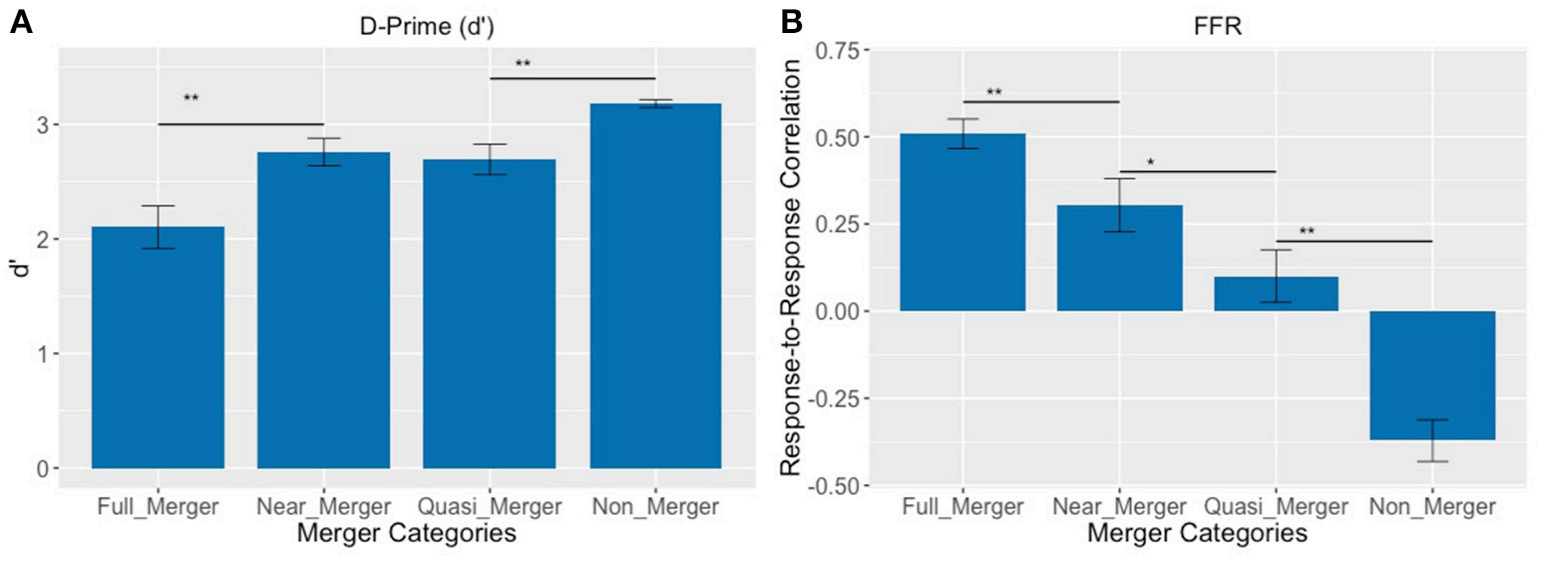
**(A)** Discrimination of tone pairs in an AX discrimination task and; **(B)** Response-to-response correlation of FFRs of tone pairs varying in their degree of tone category merging. Full-merger pair (T2/T5) showed the lowest discrimination scores and highest response-to-response correlation, whereas non-merger pair (T1/T2) showed the highest discrimination scores and lowest response-to-response correlation (^**^p < 0.01, ^*^p = 0.021; Error bars = ± SEM).

The pattern of results on d′ revealed that the subjects best discriminated the non-merger pair (T1/T2) followed by the quasi-merger pair (T3/T6), near-merger pair (T4/T6) and full-merger pair (T2/T5) (Figure 4A). There was a main effect of category on d′ with a Greenhouse-Geisser adjustment to the degrees of freedom, *F*_(2.28, 66.18)_ = 19.01, *p* <.001, $\eta_{\text{p}}^{2}$ = 0.40. Further, post-hoc paired *t*-tests revealed that participants performed better on the near-merger than full-merger pair, *t*_(29)_ = −4.33, *p* < 0.001, and better on the non-merger pair than the quasi-merger pair, *t*_(29)_ = 3.61, *p* = 0.001. However, there was no significant difference between the d′ of the near-merger and quasi-merger pairs, *t*_(29)_ = 0.69, *p* = 0.498.

#### Brainstem electrophysiology findings

**Figure 5:**
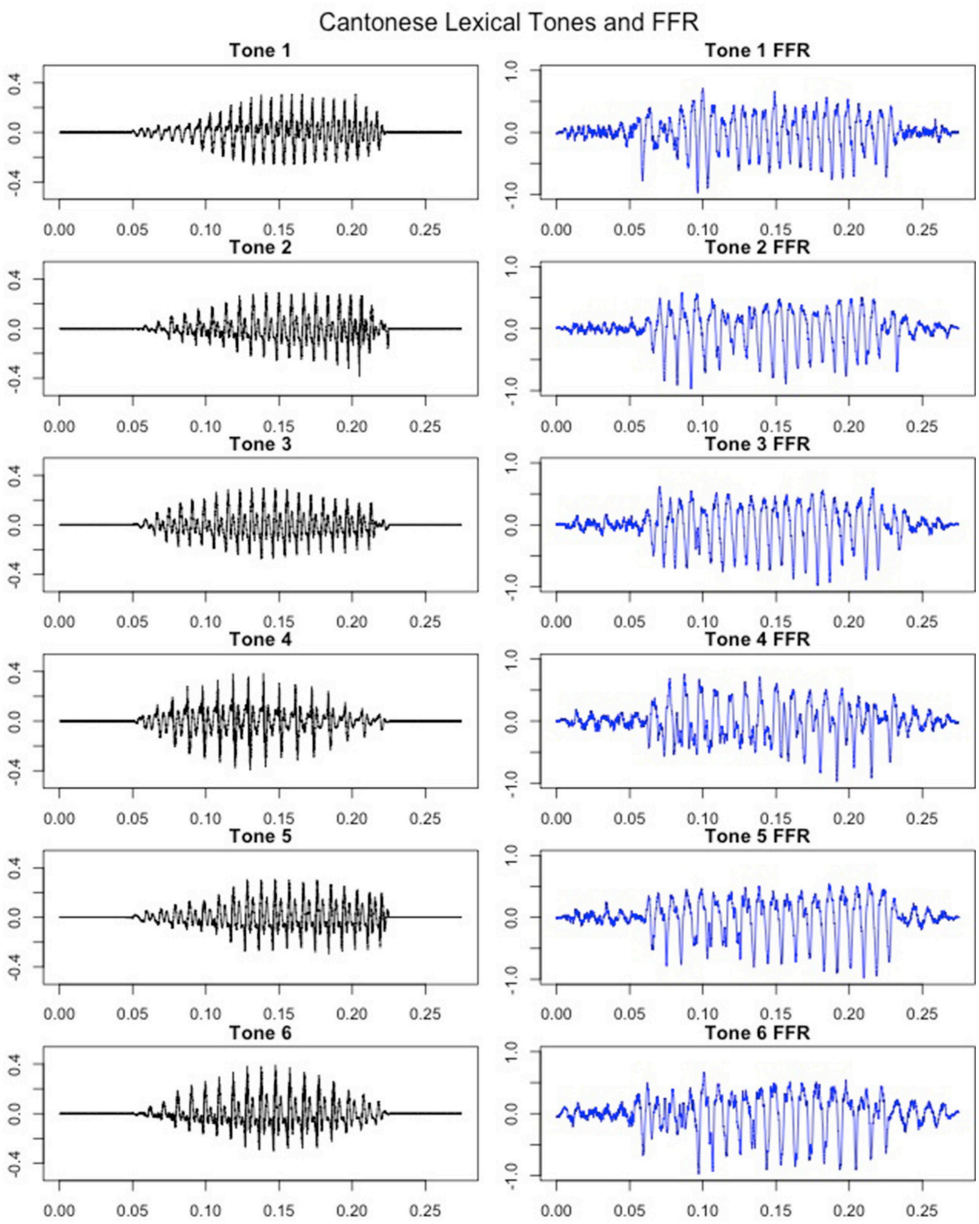
**Cantonese lexical tone stimuli (*left panel*)** and coresponding grandaveraged FFRs **(*right panel*)**. X-axis: Time (ms), Y-axis: Amplitude (Arbitrary units for stimuli in the ***left panel***; μV for FFRs in the ***right panel***). Initial and final 50 msec represents pre- and post-stimulus baselines.

FFRs were obtained for all six lexical tones of Cantonese. Figure 5 depicts waveforms from the six Cantonese lexical tones (*left panel*) and their grand-averaged FFRs (*right panel*). In order to obtain a brainstem representation of perceptual merging, response-to-response correlations were calculated for T1/T2 (non-merger), T2/T5 (full-merger), T3/T6 (quasi-merger), and T4/T6 (near-merger) tone pairs. The full-merger pair (T2/T5) showed the strongest response-to-response correlation, followed by the near-merger (T4/T6), quasi-merger (T3/T6) and non-merger (T1/T2) pairs in a sharp gradation (Figure 4B). A main effect of tone category was obtained with a Greenhouse-Geisser adjustment, *F*_(2.39, 69.3)_ = 40.09, *p* < 0.001, $\eta_{\text{p}}^{2}$ = 0.58. Further, post-hoc paired *t*-tests revealed that there was a significant difference between the full-merger pair and the near-merger pair, *t*_(29)_ = −3.41, *p* = 0.002; the quasi-merger pair and the non-merger pair, *t*_(29)_ = −4.52, *p* < 0.001, with the near- and quasi-merger pairs not reaching significance after correction for multiple comparisons, *t*_(29)_ = −2.43, *p* = 0.021.

#### Relationship between behavioral and FFR findings

**Figure 6:**
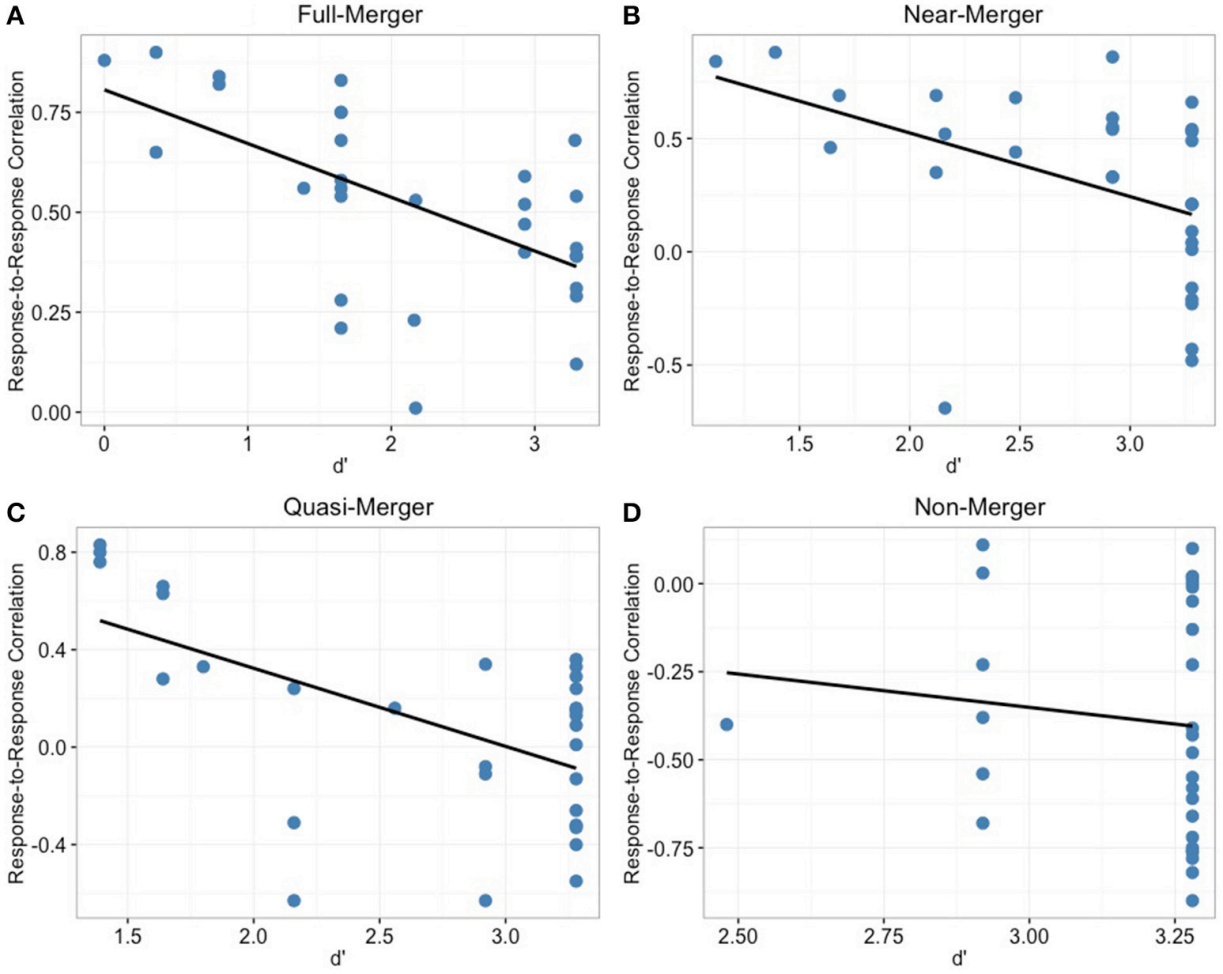
**Depicting the distribution of participants across response-to-response correlation and d′ for the four tone pair combinations**. Non-merger pair **(D)** showed no significant correlation whereas other tone pairs **(A–C)** showed significant correlations with behavioral discrimination measures.

In order to determine the relationship between behavioral discrimination abilities and brainstem measures, we calculated Pearson correlations between behavioral data (d′) of tone pairs and the corresponding response-to-response measures (Figure 6). For the non-merger (T1/T2) pair, there was no significant correlation with d′ (*r* = −0.11, *p* = 0.55). However, moderate correlations were observed between d′ and neurophysiological measures for full-merger (T2/T5) pair (d′: *r* = −0.59, *p* = 0.001), T3/T6 pair (d′: *r* = −0.57, *p* = 0.001), and near-merger (T4/T6) pair (d′: *r* = −0.44, *p* = 0.014). Musical experience did not correlate with d′ (T2/T5: *r* = 0.07, *p* = 0.722; T3/T6: *r* = 0.08, *p* = 0.678; T4/T6: *r* = 0.10, *p* = 0.593; T1/T2: *r* = 0.15, *p* = 0.435) and response-to response correlation (T2/T5: *r* = 0.05, *p* = 0.806; T3/T6: *r* = 0.07, *p* = 0.699; T4/T6: *r* = 0, *p* = 0.987; T1/T2: *r* = −0.14, *p* = 0.473).

### Experiment 2: cortex and behavior

#### Behavioral results

**Figure 7:**
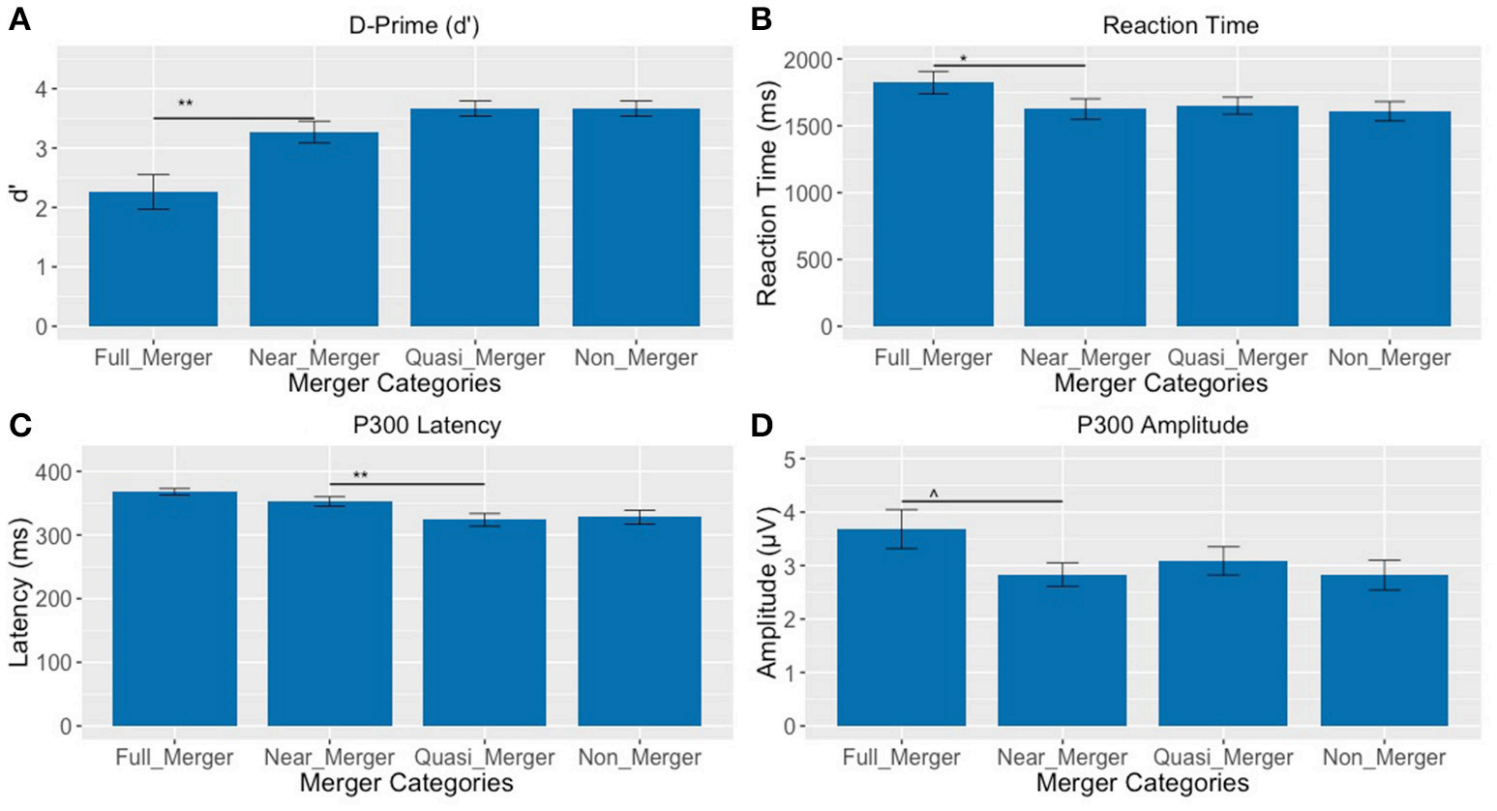
Tone pairs varying in their degree of tone category merging (^**^p <0.01, ^*^p <0.05, ^∧^*p* = 0.022; Error bars = ± SEM). (A) Non-merger pair (T1/T2) was discriminated best, whereas full-merger pair (T2/T5) was discriminated worst; **(B–D)** Full-merger pair (T2/T5) showed highest reaction time, P300 latency and amplitude, whereas non-merger pair (T1/T2) showed lowest values on these parameters.

There was a main effect of the tone categories on discrimination abilities (d′), *F*_(1.81, 43.43)_ = 14.33, *p* < 0.001, $\eta_{p}^{2}$ = 0.37. Further, post-hoc paired *t*-tests revealed a significant difference between the full-merger and near-merger pairs, *t*_(24)_ = −3.65, *p* = 0.001, but no significant difference between the non-merger and quasi-merger pairs, *t*_(24)_ = 0, *p* = 1, and quasi- and near-merger categories, *t*_(24)_ = −1.98, *p* = 0.059 (Figure 7A).

Additionally, there was a main effect of tone-categories on reaction time for discrimination, *F*_(1.95, 46.88)_ = 4.10, *p* = 0.024, $\eta_{p}^{2}$ = 0.15. Further, *post-hoc* paired *t*-tests revealed a significant difference between the full-merger and near-merger pairs, *t*_(24)_ = 2.84, *p* = 0.009, but no significant difference between the non-merger and quasi-merger pairs, *t*_(24)_ = 0.52, *p* = 0.607 or the quasi-merger and near merger pairs, *t*_(24)_ = −0.41, *p* = 0.683 (Figure 7B).

#### Cortical electrophysiology findings

**Figure 8:**
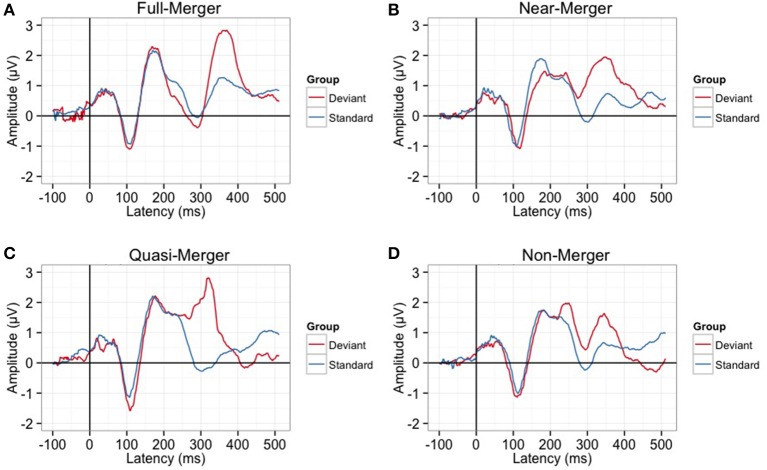
**Grand-averaged P300 waveforms for (A)** Full-merger (T2/T5) **(B)** Near-merger (T4/T6) **(C)** Quasi-merger (T3/T6) **(D)** Non-merger (T1/T2). Full-merger pair elicited the highest P300 amplitude and longest latency.

There was a main effect of the tone categories on P300 peak latency, *F*_(2.29, 55.08)_ = 7.05, *p* = 0.001. Further, *post-hoc* paired *t*-tests revealed a significant difference between the quasi- and near-merger pairs, *t*_(24)_ = −3.11, *p* = 0.005, and no significant difference between the near- and full-merger pairs, *t*_(24)_ = −2.01, *p* = 0.056 and non- and quasi-merger pairs, *t*_(24)_ = 0.29, *p* = 0.773 (Figures 7C, 8). Similarly, there was a main effect of the tone categories on P300 peak amplitude with a Greenhouse-Geisser adjustment, *F*_(3, 67.22)_ = 3.21, *p* = 0.031, $\eta_{p}^{2}$ = 0.51. Further, *post-hoc* paired *t*-tests revealed that the difference between the tone pairs did not reach significance after correction for multiple comparisons (full- and near-merger: *t*_(24)_ = −2.45, *p* = 0.022; non- and quasi-merger, *t*_(24)_ = −0.96, *p* = 0.345; quasi- and near-merger, *t*_(24)_ = 0.85, *p* = 0.404; Figures 7D, 8).

#### Relationship between behavioral and P300 findings

**Figure 9:**
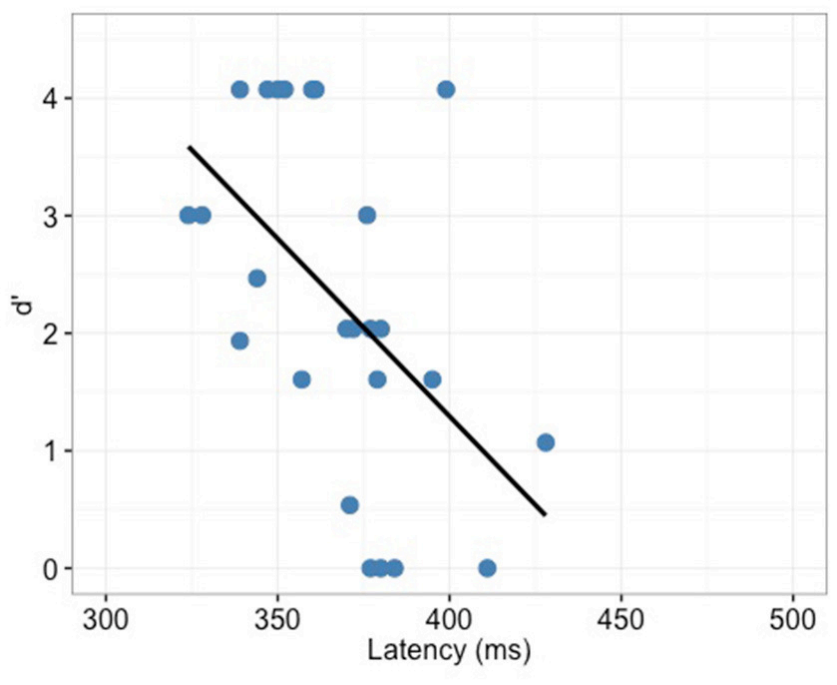
**Full-merger pair (T2/T5) showed a significant correlation (*r* = −0.52, *p* = 0.008) between d′ and P300 latency**.

P300 latency correlated significantly with the behavior (d′) only for the full-merger pair (*r* = −0.52, *p* = 0.008; Figure 9) but not with other tone pairs (quasi-merger: *r* = −0.09, *p* = 0.645; near-merger: *r* = 0.09, *p* = 0.668; non-merger: *r* = 0.26, *p* = 0.219). Additionally, no significant correlation between P300 amplitude and d′ were found (full-merger: *r* = −0.19, *p* = 0.364; quasi-merger: *r* = 0.048, *p* = 0.819; near-merger: *r* = −0.01, *p* = 0.95; non-merger: *r* = 0.25, *p* = 0.23). Musical experience did not correlate with d′ (T2/T5: *r* = 0.06, *p* = 0.766; T3/T6: *r* = −0.09, *p* = 0.684; T4/T6: *r* = −0.11, *p* = 0.603; T1/T2: *r* = −0.21, *p* = 0.331), P300 amplitude (T2/T5: *r* = −0.11, *p* = 0.595; T3/T6: *r* = 0.24, *p* = 0.255; T4/T6: *r* = −0.37, *p* = 0.071; T1/T2: *r* = −0.05, *p* = 0.807) and P300 latency (T2/T5: *r* = 0.05, *p* = 0.823; T3/T6: *r* = −0.08, *p* = 0.707; T4/T6: *r* = −0.08, *p* = 0.698; T1/T2: *r* = −0.21, *p* = 0.314).

## Discussion

In the current study, we explored sound change from behavioral and neurophysiological perspectives. Given that the Hong Kong Cantonese tone system is undergoing merging, we used it as a vehicle to study sound change. We found that there was a gradation of degree of merging across the tone categories. On average, Cantonese participants showed poorest discrimination or maximum merging for the full-merger pair (T2/T5) and best discrimination or least merging for the non-merger pair (T1/T2), while the quasi- (T3/T6) and near-merger (T4/T6) pairs showed comparable discrimination and merging. Neurophysiological measures including cortical and subcortical responses revealed a similar gradation of the tone categories. However, subcortical responses (FFR) revealed a sharper gradation or categorization than cortical neurophysiological responses and behavioral discrimination scores. Additionally, there were more individual differences in the subcortical and cortical processes for tone pairs with a greater degree of merging relative to those with less degree of merging. We propose that these individual differences can be a contributory factor to sound change.

The behavioral discrimination data are compatible with the idea that perceptual similarity/confusability leads to sound change (Ohala, 1981, 2012). Based on reaction time data in an AX discrimination paradigm, (Mok et al., 2013) found that their participants took longer on T2/T5 discrimination than T1/T2 discrimination. Recently, there have been studies (Law et al., 2013) classifying participants into merger and/or non-merger groups based on their speech production and/or perception. The literature on Cantonese tone merging has been limited to studying merging as a discrete phenomenon. However, in order to systematically investigate sound change in Cantonese, tone merging must be viewed as a continuum across the tone categories. In the present study, we evaluated one non-merging pair and three merging pairs of Cantonese lexical tones that varied in their degrees of merging. The current study, for the first time investigated the sound change as a continuum of Cantonese tone merging.

Individual variability is considered to be an important factor for dissemination of variants of sound change (Witkin et al., 1975; Messick, 1976; Ausburn and Ausburn, 1978; Yu, 2010, 2013). In the current study, variability in speech perception across individuals was established behaviorally and neurophysiologically. Significant correlations were found between behavioral discrimination scores and FFR response-to-response correlations for the tone merging pairs, whereas there was no correlation between the behavioral and neurophysiological aspects of the non-merger pair. Similarly, there was a significant correlation between the behavioral discrimination scores for the full-merger pair and corresponding P300 latency, whereas there were no significant correlations in the lesser or non-merging pairs. The absence of reliable correlations between behavioral and neurophysiological measures for the lesser or non-merging pairs and the presence of reliable correlations for the merging pairs confirms that the individual variability among listeners is equally well-represented in their neurophysiology and behavior, and may in turn contribute to sound change. Thus, the findings from the present study establish a plausible link between sound change, behavior and neurophysiology.

In the FFR data, stronger response-to-response correlations suggested more merging of tone categories in the brainstem. In the cortical neurophysiological data, higher values of amplitude and latency corresponded with increased merging of tones in the tone pairs. A sharper gradation of the merging phenomenon was evident in the brainstem as compared to cortical and behavioral data, probably because of sharp phase locking abilities of the brainstem. Recently, Law et al. (2013) conducted an ERP study exploring tone merging at cortical levels using MMN and P3a. They found that individuals who performed poorly on T4/T6 (near-merger) discrimination tasks had a smaller MMN and P3a amplitude. However, as merger pairs with varying degrees of merging were not included, their study was limited to investigating merging as a discrete phenomenon and not as a continuum to account for sound change. In the current study, instead of looking at inattentive levels of processing using MMN or P3a, we looked at attentive levels using P300. Additionally, a P300 task challenges the capacities of the auditory and attention system as a whole and reflects the effort devoted by individuals in discriminating stimuli (Luck, 2005). Therefore, use of a P300 task, as in the current study, is informative of active language processing and a better indicator of tone merging.

The source of individual variability in the behavioral and neurophysiological findings of the current study can be discussed in light of bottom-up processing (Krishnan et al., 2005, 2009, 2010), top-down processing (Suga, 2008; Parbery-Clark et al., 2009; Krizman et al., 2012; Song et al., 2012; Hairston et al., 2013; Skoe and Kraus, 2013), and the predictive tuning hypothesis (Chandrasekaran et al., 2014). The brainstem has been found to be sensitive to language experience as lexical tones are more accurately represented in tone language speakers than non-tone language speakers. Krishnan et al. (2005, 2009, 2010) argue that the experience-dependent effects in brainstem responses are due to local reorganization of brainstem circuits via excitatory and inhibitory synaptic plasticity. This account has been supported by animal models where it was found that auditory midbrain neurons rapidly adapt to the dynamic stimulus characteristics (Escabí et al., 2003; Dean et al., 2005; Pérez-González et al., 2005; Dahmen et al., 2010). In other words, the auditory brainstem is locally malleable. In the current study, the participants who may be inducing variability (called *innovators*) may be the ones with aberrant encoding of lexical tones at the level of the inferior colliculi. As a result of this, there is an inefficient transmission of information to cortex that is leading to deficient perception.

Recently, it has also been found that the effects visible at the level of the brainstem originate at the level of the cortex and are driven via corticofugal pathways from the cortex to the brainstem (Suga, 2008; Parbery-Clark et al., 2009; Bajo et al., 2010; Krizman et al., 2012; Song et al., 2012; Hairston et al., 2013; Skoe and Kraus, 2013). This view has gained support from animal models where inactivation of auditory cortex has led to disruption of brainstem plasticity (Suga et al., 2000, 2002; Zhou and Jen, 2000; Suga, 2008). Gao and Suga (1998) found that behaviorally relevant sounds reflect more activity in the inferior colliculi as compared to acoustic stimulation alone. Tone merging originating in the cortex, as observed in the current study, could have driven merging at the level of the brainstem. We found that the tone pairs showed similar trends of merging in brainstem and cortical measures. The pairs with longer P300 latencies also showed stronger response-to-response correlations, and vice versa. These findings show that the individuals inducing variability might have learned the pitch contours of the lexical tones in a divergent manner. Learning could have led to divergent cortical changes. Corticofugal effects of this divergent experience-dependent learning could have led to deviant representations in the brainstem. Though, both cortical and brainstem mechanisms may be affected with divergent learning, the effects are more well-defined and represented in the brainstem. This could be due to more accurate phase locking and fidelity of representation of lexical tones in the brainstem.

The findings of the current study can also be explained using the predictive tuning model (Chandrasekaran et al., 2014) which proposes that there is continuous, online modulation of brainstem encoding by the auditory cortex via corticofugal pathways (Chandrasekaran et al., 2009a; Chandrasekaran and Kraus, 2010) along with local processes in the inferior colliculi still being active. Signal representation gets enhanced at each level when the stimulus matches the expectation or prediction from a higher level. In the current study, it is possible that individuals contributing to variability (innovators) are not able to predict the F0 contour of the lexical tones accurately and thus are matching to a slightly deviant representation at the cortical levels as a result of which there is enhancement of slightly deviant representation at the brainstem level.

Though the results of the current study provide an insight into the subcortical and cortical neural representations of sound change in a group of Cantonese listeners, determining a causal relationship between the two is beyond the scope of this study. The results from the current study have implications toward studying atypical populations (e.g., autism) with cognitive and/or perceptual aspects deviant to the typical population, in order to investigate the neurophysiological indicators of sound change in greater detail. Future studies could use the latest pitch-specific ERP techniques such as cortical pitch response (Krishnan et al., 2012, 2014, 2015, 2016) to probe sound change in tone languages. Further, as other linguistic factors such as frequency of occurrence have been known to affect sound change (Hooper, 1976; Pierrehumbert, 2001; Abramowicz, 2007; Zhao and Jurafsky, 2009), future studies may conduct an in-depth investigation of neurophysiological indicators of sound change from the perspective of frequency of usage and exemplar theory (Abramowicz, 2007). Concomitantly, the data from the current study also exhibited a lack of influence of musical training on behavioral and neurophysiological differentiation of lexical tone contrasts. For investigating the key mechanisms responsible for these findings, another study from our group is underway.

## Conclusions

In the current study, we investigated the contribution of individual variability toward sound change in Hong Kong Cantonese. We found that the sound change in Cantonese, indicated by reshaping of tone categories was observable at the levels of the brainstem and cortex. Moreover, we found that the newly merging categories that are not yet clearly expressed at the behavioral and cortical levels appear delineated at the subcortical level. These findings have implications toward the active contribution of the brainstem in driving sound change. Overall, we speculate that the individuals with divergent encoding of speech sounds in their subcortical and cortical processes might produce speech in a deviant manner that spreads across the community and leads to sound change over a period of time. However, a causal relationship between the neural representations and sound change still remains an open question.

## Ethics statement

The Joint Chinese University of Hong Kong—New Territories East Cluster Clinical Research Ethics Committee. Written informed consents were obtained from all participants prior to the experiments. The Joint Chinese University of Hong Kong—New Territories East Cluster Clinical Research Ethics Committee approved the study.

## Author contributions

AM: Data collection, Experiment designing (Behavioral and EEG), Data analysis, Manuscript writing. FL: Experiment designing (Behavioral), Data collection and analysis. MA: Data analysis, Manuscript writing. PW: Experiment designing (Behavioral and EEG), Data analysis, Manuscript writing.

### Conflict of interest statement

The authors declare that the research was conducted in the absence of any commercial or financial relationships that could be construed as a potential conflict of interest.
