# Supplementary material for: Neural Correlates of Indicators of Sound Change in Cantonese: Evidence from Cortical and Subcortical Processes
Source: Front Hum Neurosci. 2016 Dec 23;10:652. doi: 10.3389/fnhum.2016.00652 (PMC5179532; doi:10.3389/fnhum.2016.00652)
Supplement: Supplementary file 1 [file DataSheet1.doc]

Supplementary Material

**Neural correlates of indicators of sound change in Cantonese: Evidence from cortical and subcortical processes**

**Akshay Raj Maggu^1^, Fang Liu^2^, Mark Antoniou^3^, Patrick C. M. Wong^1,4,5*^**

^1^Department of Linguistics and Modern Languages, The Chinese University of Hong Kong, Shatin, N.T., Hong Kong SAR, China

^2^School of Psychology & Clinical Language Sciences, University of Reading, Reading, UK

^3^The MARCS Institute for Brain, Behaviour and Development, Western Sydney University, Locked Bag 1797, Penrith NSW 2751, Australia

^4^Brain and Mind Institute, The Chinese University of Hong Kong, Shatin, N.T., Hong Kong SAR, China

^5^The Chinese University of Hong Kong – Utrecht University Joint Center for Language, Mind and Brain

***Correspondence:**Patrick C. M. Wong, Department of Linguistics and Modern Languages, The Chinese University of Hong Kong, Room G03, Leung Kau Kui Building, Shatin, N.T., Hong Kong, China.

p.wong@cuhk.edu.hk

Supplementary Table 1. Correlation matrix of stimulus-to-stimulus correlation for all tone-pair combinations

|  | Tone 1 | Tone 2 | Tone 3 | Tone 4 | Tone 5 | Tone 6 |
| --- | --- | --- | --- | --- | --- | --- |
| Tone 1 | 1 |  |  |  |  |  |
| Tone 2 | -.64 | 1 |  |  |  |  |
| Tone 3 | .48 | -.69 | 1 |  |  |  |
| Tone 4 | .72 | -.98 | .69 | 1 |  |  |
| Tone 5 | -.27 | .63 | -.41 | -.66 | 1 |  |
| Tone 6 | .53 | -.91 | .64 | .93 | -.76 | 1 |

Supplementary Table 2. Correlation matrix of response-to-response correlation for all tone-pair combinations. Each cell contains Mean and Standard Error (in parentheses)

|  | Tone 1 | Tone 2 | Tone 3 | Tone 4 | Tone 5 | Tone 6 |
| --- | --- | --- | --- | --- | --- | --- |
| Tone 1 | 1 |  |  |  |  |  |
| Tone 2 | -.38 (.05) | 1 |  |  |  |  |
| Tone 3 | .01 (.08) | .06 (.09) | 1 |  |  |  |
| Tone 4 | .21 (.08) | -.45 (.09) | -.08 (.09) | 1 |  |  |
| Tone 5 | -.34 (.10) | .52 (.04) | .25 (.10) | -.42 (.07) | 1 |  |
| Tone 6 | .16 (.10) | -.51 (.09) | .10 (.07) | .31 (.08) | -.42 (.05) | 1 |


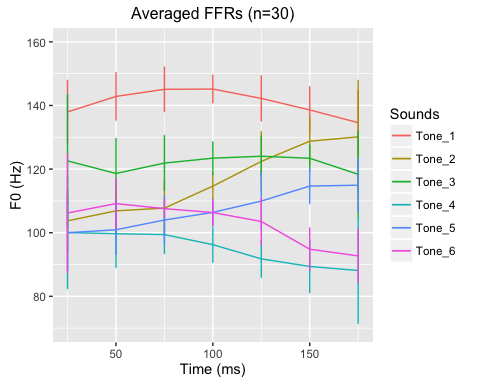


Supplementary Figure 1. F0 contours of average FFRs (n=30) for the six lexical tones of Cantonese. (Error bars = ± S.D.)
